# Division of Labor in Colonies of the Eusocial Wasp, *Mischocyttarus consimilis*

**DOI:** 10.1673/031.012.2101

**Published:** 2012-02-09

**Authors:** Viviana O. Torres, Thiago S. Montagna, Josué Raizer, William F. Antonialli-Junior

**Affiliations:** ^1^ Laboratório de Ecologia, Centro Integrado de Análise e Monitoramento Ambiental, Universidade Estadual de Mato Grosso do Sul, 351, 79804-907, Dourados-MS, Brazil; ^2^ Faculdade de Ciências Biológicas e Ambientais, Universidade Federal da Grande Dourados, 241, 79804-970, Dourados-MS, Brazil

**Keywords:** age, division of tasks, dominance, ethogram, Polistinae

## Abstract

The division of labor between castes and the division of labor in workers according to age (temporal polyethism) in social wasps are crucial for maintaining social organization. This study evaluated the division of labor between castes, and the temporal polyethism in workers of *Mischocyttarus consimilis* Zikán (Hymenoptera: Vespidae). To describe the behavioral repertory of this species, observations were made of 21 colonies, with 100 hours of observations. In order to observe temporal polyethism, each newly emerged wasp was marked with colored dots on the upper area of the thorax. This allowed the observation of behavioral acts performed by each worker from the time of emergence to its death. Through hybrid multidimensional scaling, a clear division between queens and workers could be identified, in which the behaviors of physical dominance and food solicitation characterized the queen caste; while behaviors such as adult—adult trophallaxis, destruction of cells, alarm, foraging for prey, foraging for nectar, and unsuccessful foraging characterized the worker caste. Hybrid multidimensional scaling characterized two groups, with intra—nest activities preferentially accomplished by younger workers, while extra—nest activities such as foraging were executed more frequently by older workers.

## Introduction

Eusocial wasps, including some species of Stenogastrinae and all members of Polistinae and Vespinae, are characterized by overlapping adult generations, reproductive division of labor, and cooperative brood care (Wilson 1971; Michener 1974). New colonies are established by independent foundation or by swarming. Wasps of the tribes Polistini and Mischocyttarini and some species of Ropalidiini adopt independent foundation, with one (haplometrosis) or more (pleometrosis) females beginning the construction of the nest, while members of the tribe Epiponini begin new colonies by swarming (Von Ihering 1896; Jeanne 1980).

In wasps with independent foundation, there is no morphological caste differentiation, and the caste of queen or worker is determined mainly by means of aggressive interactions (Gadagkar 1991). The queens, being more aggressive, can ingest more food during trophallaxis (Jeanne 1972; Röseler 1991; Spradbery 1991), which may cause them to develop their ovaries (Queller and Strassmann 1989). However, the determination of castes can occur during the pre—imaginal phase (Hunt 1991; O'Donnell 1998a), affecting the larval nutrition and the rate of development of the immatures, and consequently the division of castes (West-Eberhard 1969; Gadagkar et al. 1991; O'Donnell 1998a). Pre—imaginal determination of caste is evident in swarm— founding species with morphologically distinct castes, including some Epiponini and Ropalidiini (Jeanne 1991a), and also in some species with independent foundation, such as *Ropalidia marginata* (Gadagkar et al. 1991), *Belonogaster petiolata* (Keeping 2002), and other members of *Polistes* (O'Donnell 1998a).

Other investigators, for example Brillet et al. (1999), have suggested that in some wasp species such as *P. dominula*, the abdominal vibrations produced by the founders may influence the future status of newly emerged wasps, determining which will become workers or future founders. Sound production during the larval feeding of eusocial wasps was observed by Pratte and Jeanne (1984) and was described as antennal drumming. In recent studies, Jeanne (2009) and Suryanarayanan et al. (2011) suggested that the vibrational signals in the nest may affect caste development, by means of biochemical changes and gene expression at the larval stage.

After establishing the colony, the queen must maintain her hierarchy. Early studies indicated that maintaining the dominance hierarchy in colonies of less derived wasps was determined by the presence of the queen laying in the nest, caring for the brood, and attacking other females (Strassmann 1985; Jeanne 1972). The queens avoid activities with a high risk of predation and high energy cost, such as foraging, which are carried out more frequently by workers (Strassmann et al. 1984; O'Donnell 1998a). Therefore the behavioral repertory of workers is generally broader, such as in *P. dominula* (Pardi 1948), *Mischocyttarus drewseni* (Jeanne 1972), *M. cerberus styx* (Giannotti 1999), *P. versicolor* (Zara and Balestieri 2000), and *P. canadensis canadensis* (Torres et al. 2009).

The division of non—reproductive tasks among nestmates, or polyethism, is one of the largest evolutionary advantages that led to the ecological success of the social insects (Hölldobler and Wilson 1990). Dominance interactions in determining reproductive status in the polistine eusocial wasps (Pardi 1948; West-Eberhard 1969) can also structure polyethism in several species (Reeve and Gamboa 1987; Jeanne 1991a; O'Donnell and Jeanne 1995; O'Donnell 1998b, 1998c).

In eusocial Hymenoptera, the division of labor between queens and workers usually increases with the size of the colony (Jeanne 1986), and the degree of temporal polyethism varies with species and also seems to be related to colony size (Wilson 1971; Jeanne 1999). In colonies of ants, bees, wasps, and termites with thousands to millions of individuals, there is a clear division of tasks, and workers are highly specialized (Jeanne 1986, 2003; Hölldobler and Wilson 1990). Body size and colony composition are better correlated with the behavioral changes of individuals in the transition from intra—nest to extra—nest tasks, such as foraging activities (Free 1955; Cameron 1989; Röseler and Van Honk 1990). The development of behavioral specialization in the colony may be related to colony expansion, and consequently to the increase in demanding tasks (Gaustrais et al. 2002). Hence, in small colonies with fewer than a hundred individuals, individuals were observed performing a wide variety of tasks (Traniello 1978; Jeanne 1991b; Karsai and Wenzel 1998), which indicates the existence of high behavioral plasticity.

*Mischocyttarus* consimilis Zikán (Hymenoptera: Vespidae) is an independent— founding eusocial Neotropical wasp. This species was formerly restricted to Paraguay, but has recently dispersed through southern Mato Grosso do Sul and western Paraná states in Brazil. Because of this restricted distribution, the species has been the subject of few studies except those by Montagna et al. (2009, 2010) and Torres et al. (2011). In addition, Montagna and coworkers have recently observed and studied the first case of facultative parasitism in the genus *Mischocyttarus*, the congener *M. cerberus.* In view of these features, the aim of this study was to investigate the division of labor in colonies of *M. consimilis*, in order to better understand aspects related to the evolution of social behavior in wasps.

## Materials and Methods

Observations were carried out on *M. consimilis* colonies under natural conditions. The nests were constructed on buildings at the Universidade Federal da Grande Dourados (UFGD) in Mato Grosso do Sul, central— western Brazil (22° 13′ 16″ S, 54° 48′ 20″ W).

### Division of labor between castes

Observations were carried out on 21 colonies between May and December of 2007, in order to investigate the division of labor between the castes. Four of the colonies were in the pre—emergence stage and were pleometrotic foundations in which the queen had already established her status, 14 were in the post— emergence stage, and three were in decline. Therefore, the observations were made at different stages of the colonial cycle. The classification of colony developmental stages followed the methodology proposed by Jeanne (1972).

To determine the behavioral repertory of the species, 20 hours of qualitative observations in sessions of 60 min each were carried out. The method of all occurrences (“*ad libitum*” sensu Altmann 1974) was used, which involves observing the animal's behavior including its entire performance, i.e., its movements and/or immobility. The categories and behavioral acts of both castes were defined by this method. These qualitative observations were performed for eight colonies. Four colonies in post—emergence were observed for 10 hours, two colonies in decline for five hours, and two colonies that were in pre—emergence and were founded by pleometrosis were observed for five hours.

Following this stage, 80 hours of quantitative monitoring were carried out in 60—min sessions three times a week. During the sessions, five—min observations were made, with two—min intervals by the scanning method (“scanning sample” sensu Altmann 1974). Behaviors performed by all individuals in the colony were observed, totaling 7500 behavioral records.

For identifying queens and workers in colonies of *M. consimilis*, parameters related to the position and behaviors of wasps were used, as described by Jeanne (1972), Giannotti (1999), Giannotti and Machado (1999), and Torres et al. (2009), in which the queen remains longer in the nest center, is the main egg—layer, is the more aggressive female, and starts either most of the cells or all of them. On the other hand, the workers perform other activities such as colony maintenance and foraging, and are submissive to the queen.

**Figure 1.**
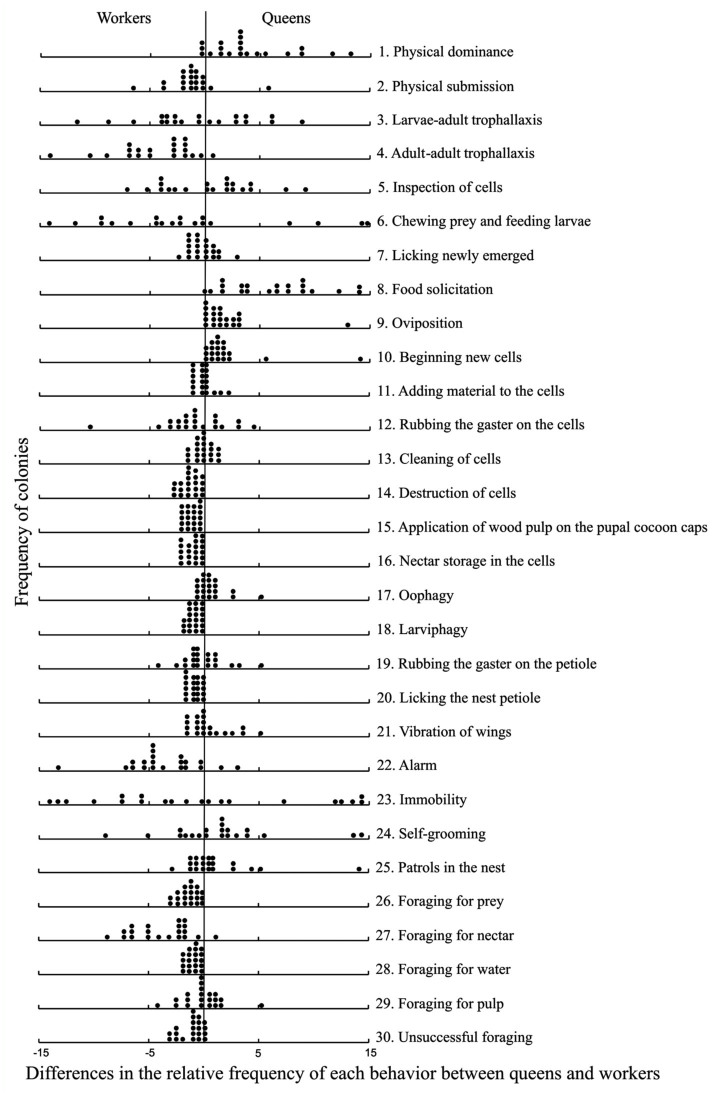
Differences in the relative frequency of records of each behavior between queens and workers in 21 colonies of *Mischocyttarus consimilis.* Each dot represents a colony. Positive values (right of the vertical line) indicate higher frequency of the behavior in queens, and negative values (left of the vertical line) indicate higher frequency of the behavior in workers. High quality figures are available online.

The behaviors were not described individually, since the behavioral repertory (Figure 1) is similar to descriptions in previous studies, such as that of Jeanne (1972) with *M. drewseni*, Giannotti (1999) with *M. cerberus styx*, Giannotti and Machado (1999) with *Polistes lanio*, Zara and Balestieri (2000) with *P. versicolor*, and Torres et al. (2009) with *P. canadensis canadensis.* However, definitions of those behaviors in which the meaning is vague are described below in Figure 1.

**Physical dominance.** A wasp grabs and pulls with their mandibles the legs, wings, and/or the antennae of the other in order to immobilize it. Two or more wasps can attack a single individual.

**Physical submission.** Passively enduring an aggression from one or more wasps at the same time, generally with their body close to the nest surface, and/or often moving away from the aggressor(s), being able to fly from the nest.

**Alarm.** Moving the gaster, shrinking and expanding it, flickering its wings and pointing the antennae and head out to the source of disturbance. They can also beat their first pair of legs in the cells of the nest, flicker their wings, and attack the source of disturbance.

**Rubbing the gaster on the cells.** Moving the gaster by rubbing it from one side to the other at the cells extremities every time a forager lands in the nest.

**Inspection of cells.** Inserting the head inside the cells, hitting their antennae inside the walls, producing sometimes inaudible sound, similar to that described by Pratte and Jeanne (1984). This is common during larval feeding. Furthermore, during this behavior, the wasp can move its gaster rapdily up and down, flexing it.

The identification of the type of material collected by foragers (water, nectar, wood pulp, prey, or unsuccessful) was based on the behaviors performed by foragers as they returned to the nest, according to methodology used by Montagna et al. (2009), who conducted a study of foraging activity in this species. Individuals that flew from the nest for a few seconds and then returned were not taken into account.

The behavioral differences were analyzed based on the relative frequency of each behavioral act performed by queens and workers in the 21 colonies of *M. consimilis.* In Figure 1, each dot represents a colony and the positive values indicate a higher frequency of the behavior for the queen caste, and negative values indicate a higher frequency for the worker caste. The number of wasps varied according to the stage and size of the colony and the observation period, but did not reach more than 16 individuals in the post— emergence stage (Torres et al. 2011). Hybrid multidimensional scaling (HMDS) was used (Belbin 1991; Faith et al. 1987) to obtain a representative gradient of behavioral variation between workers and queens. A Bray-Curtis dissimilarity matrix was used to perform this HMDS ordination, based on relative frequencies of behavior for the castes of each colony. To define whether the observed behavioral pattern differed between the two castes, a linear model was used in which the gradient obtained by the ordination (behavioral pattern) is the dependent variable, and the caste type is the independent variable; the multivariate criteria test statistic Pillai's Trace was used, following Legendre and Legendre (1998) and Borcard et al. (2011).

In computer simulations, Faith et al. (1987) demonstrated that the behavioral differences are best represented by the Bray—Curtis distance. Therefore, the HMDS method proposed by Belbin (1991) was chosen, which allows the use of any measure of difference between samples (as in any method of multidimensional scaling) and does not assume linear relationship between attributes.

### Temporal polyethism

Temporal polyethism was analyzed based on observations carried out from January to July 2008 on five colonies all in post—emergence. The five largest colonies were selected because more offspring were available for behavioral comparison. During the observations, 97 workers were monitored. Each emerged individual was marked with one or more colored dots of nontoxic ink on the upper area of the thorax, similar to the method used by Nakata (1996). This procedure allowed behavioral observation and behavior frequencies for the lifetime of every marked worker since its emergence.

Colonies were monitored in 60—min sessions three times a week. During the sessions, five— min observations were made, with two—min intervals to quantify the behavioral acts executed by each marked individual (Altmann 1974). Age intervals were established following the method used by Giannotti and Machado (1994); however, five—day intervals were used, because 21 workers began to forage in the first week of adult life, differing from *P. lanio* (Giannotti and Machado 1994), in which the workers began foraging activity only after the first week of life. The observations were performed from emergence to permanent disappearance from their nest, which was assumed to be caused by death.

To obtain a representative gradient of the behavioral variation among workers, 97 workers were ordered by HMDS (Belbin 1991; Faith et al. 1987). The Bray—Curtis dissimilarity was calculated from a frequency matrix of each behavior. To assess whether the behavioral pattern differed among workers of different ages, a general linear model was used, in which the gradient obtained by the ordination (behavioral pattern) is the dependent variable, and the age interval is the independent variable. Pillai's Trace was used according to Legendre and Legendre (1998) and Borcard et al. (2011).

## Results

### Division of labor between castes

30 behavioral acts were observed in the colonies of *M. consimilis* (Figure 1). 23 were executed by queens and 29 by workers; 22 were common to both castes. Among all the behaviors, only the act of beginning new cells was exclusive to the queen. The workers showed seven exclusive acts: destruction of cells, application of wood pulp on the pupal cocoon caps, nectar storage in the cells, larviphagy, licking the nest petiole, foraging for prey, and foraging for water; each of these related to the maintenance of the colony.

Licking the nest petiole behavior did not differ significantly among the castes, although it occurred more frequently in the workers (Figure 1). The physical dominance behaviors, food solicitation, and oviposition were more often executed by the queens, whereas the behaviors of adult—adult trophallaxis, alarm, foraging for nectar, and unsuccessful foraging were more frequently executed by the workers (Figure 1).

**Figure 2.**
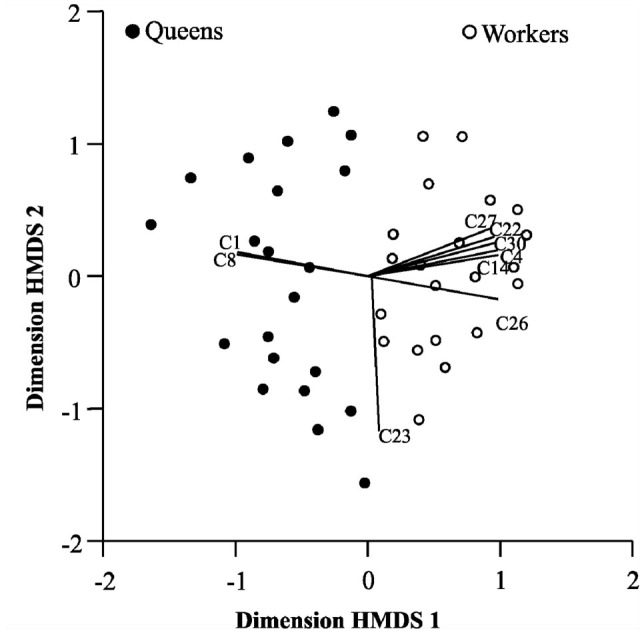
Analysis of division of labor between queen and worker castes by means of ordination by hybrid multidimensional scaling, in two dimensions (stress = 0.25), of the nests of *Mischocyttarus consimilis* (21 records for queens, filled circles; 21 records for workers, empty circles) according to the 30 defined behavioral acts. The vectors represent the behavioral acts that contributed most to the ordination (r > 0.5). Cl = physical dominance, C8 = food solicitation characterized the repertory of queens, C4 = adult—adult trophallaxis, C14 = destruction of cells, C22 = alarm, C26 = foraging for prey, C27 = foraging for nectar, C30 = unsuccessful foraging characterized the workers' repertory, C23 = immobility was common to both castes. High quality figures are available online.

A clear division between the behaviors performed by workers and queens was apparent from the sample ordination by HMDS (Figure 2). The right side showed a more significant performance by the workers, and the left a more significant performance by the queens. Caste significantly explained the ordination pattern (Pillai's Trace = 0.736; *F* = 54.355; gl= 2 and 39; *p* < 0.01). As seen in Figure 2, the behaviors that contributed most to separate the two castes were: physical dominance (C1) and food solicitation (C8), which characterized the repertory of the queens; and adult—adult trophallaxis (C4), destruction of cells (C14), alarm (C22), foraging for prey (C26), foraging for nectar (C27), and unsuccessful foraging (C30), most of these related to the maintenance of the colony, characteristic of worker repertory. Immobility (C23) was common to both castes.

### Temporal polyethism

**Figure 3.**
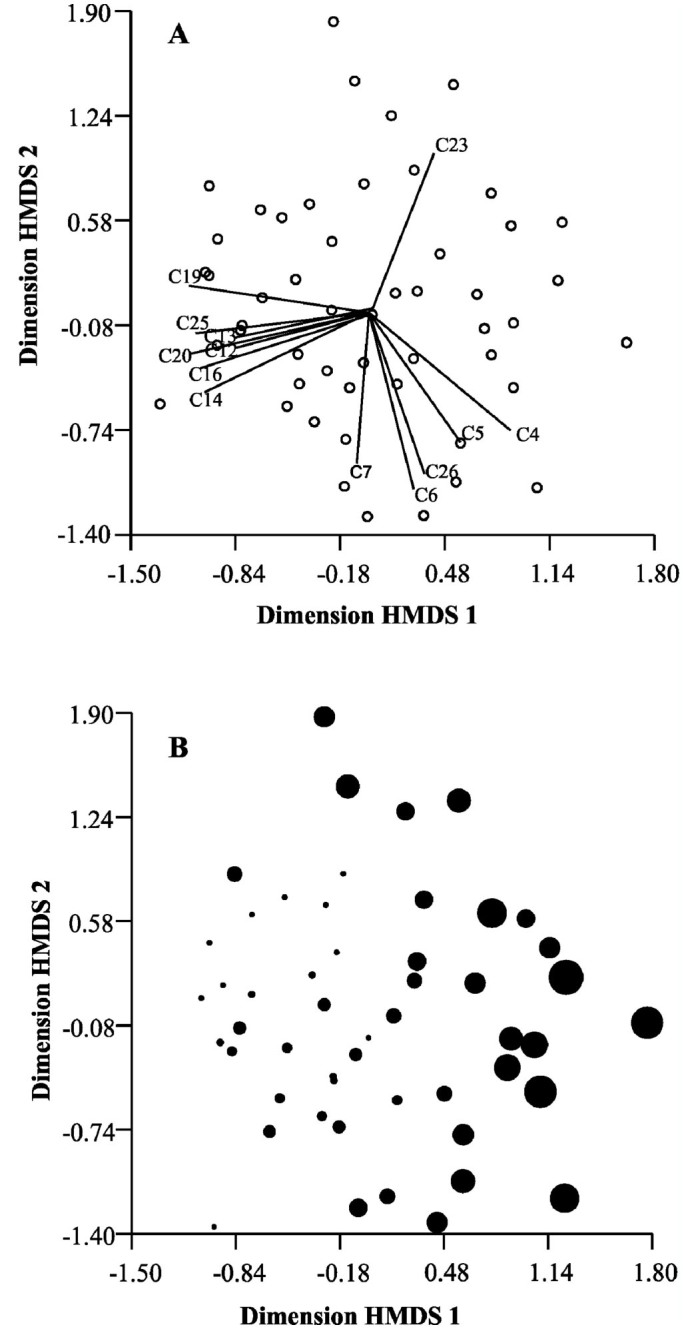
Analysis of temporal polyethism of workers of *Mischocyttarus consimilis* in five colonies, by ordination by hybrid multidimensional scaling in two dimensions (stress = 0.27). In A the vectors represent the relative contribution of each behavior to the plan of the ordination seen in B (r > 0.5). In B, the size of the points is directly proportional to the workers' age. C4 = adult-adult trophallaxis, C5 = inspection of cells, C6 = chewing prey and feeding larvae, C7 = licking newly emerged, C12 = rubbing the gaster on the cells, C13 = cleaning of cells, C14 = destruction of cells, C16 = nectar storage in the cells, C19 = rubbing the gaster on the petiole, C20 = licking the nest petiole, C23 = immobility, C25 = patrols in the nest, C26 = foraging for prey. High quality figures are available online.

Behavioral pattern obtained from the HMDS for temporal polyethism shown by the workers can be represented in two dimensions (Figure 3). This ordination indicated a gradient in which some behaviors are executed more frequently by younger workers, while others are more common in older workers (Figures 3a, 3b). In the multivariate analysis, age significantly explained the pattern of behavioral variation (obtained by HMDS) of the worker caste (Pillai—Traces = 0.806; *F* = 2.195; gl = 24 and 78; *p* < 0.01).

The mean longevity of workers was 24.26 ± 10.68 days (4–77, n = 97). They began to forage at a mean age of 8.54 ± 3.24 days (1– 20, n = 97); however, 21 wasps began foraging in the first week of life.

The behaviors of rubbing the gaster on the cells, cleaning of cells, destruction of cells, nectar storage in the cells, rubbing the gaster on the petiole, licking the nest petiole, and patrols in the nest were executed more frequently by younger workers and are related to colony maintenance, indicating that the younger individuals remain in the nest most of the time (Figures 3a, 3b).

The behaviors of adult—adult trophallaxis, inspection of cells, chewing prey and feeding larvae, and foraging for prey were executed more frequently by older workers, indicating that these workers spend more time in activities that require more energy and involve a greater risk of predation (Figures 3a, 3b).

## Discussion

A clear division of labor between queens and workers in colonies of *M. consimilis* was apparent. Queens spent longer periods of time in the nest, devoting their time to activities of dominance hierarchy and oviposition, while workers were engaged more frequently in maintenance activities, as well as the defense and success of the colonies. This species does not exhibit a well—defined temporal polyethism. However, in general, younger workers spend more time in intra—nest activities and older workers perform more extra—nest activities. These two groups may, if necessary, overlap their repertory, demonstrating a typical behavioral plasticity that occurs in this group of less derived social wasps.

The behaviors of physical dominance, food solicitation, and oviposition were executed more often by the queens as also observed in colonies of *M. c. styx* (Giannotti 1999), *P. lanio* (Giannotti and Machado 1999), and *P. versicolor* (Zara and Balestieri 2000). According to Oliveira et al. (2006), dominance interactions and subordination are increased in large colonies and during post— emergence.

Similarly to *M. consimilis*, in the colonies of *M. drewseni*, the queens perform most of the ovipositions (Jeanne 1972). This differs from *P. lanio*, in which oviposition was done exclusively by the queens, confirming the condition of functional monogyny in nests (Giannotti and Machado 1999). According to Deleurance (1950), the presence of empty cells stimulates oviposition in *Polistes*, and the queen, by maintaining the cells full of her own eggs, prevents the workers from ovipositing (Brian 1958).

On the other hand, the behaviors of adult— adult trophallaxis, alarm, foraging for prey, foraging for nectar, and unsuccessful foraging were carried out more frequently by the workers, as also observed by Giannotti (1999). As West-Eberhard (1969) described, during trophallaxis between adults, it was possible to detect a difference between the donor's posture and that of the receiver. The alarm behavior occurred significantly more often in workers, showing that this behavior is important for nest defense. Several studies have shown that during this act, the wasp releases certain volatile substances that function as an alarm pheromone, recruiting nestmates and motivating an attack on the source of disturbance (Ishay 1965; Jeanne 1982; Ono et al. 2003; Fortunato et al. 2004).

Concerning the temporal polyethism of *M. consimilis*, intra—nest tasks such as caring for the offspring are more frequently carried out by younger workers. The high—risk tasks such as foraging and defense of the nest are done by older workers, as occurs in several species of social wasps (West-Eberhard 1996). This division of tasks between older and younger workers occurs through genetic predetermination (Page and Robinson 1990; O'Donnell 1996), and according to the conditions of the colony such as the size and age of the offspring, damage to the nest, the presence of predators and parasites, and the size and age of the worker population (Wilson 1971; O'Donnell and Jeanne 1992; Inoue et al. 1996; Naug and Gadagkar 1998).

On the other hand, this pattern, as previously described for *M. mastigophorus*, can be affected by queen—worker and worker—worker interactions (O'Donnell 1998c). The queens of species that show independent foundation act as the main precursors, behaviorally regulating the tasks to be accomplished by the workers (Reeve and Gamboa 1987; Gamboa et al. 1990). However, in some species, the dominance interaction among workers can induce foraging activity by other workers (Premnath et al. 1995; O'Donnell 1998a). Therefore, the dominance behavior among workers can play a role in structuring polyethism, even though these workers have little effect on the reproductive competition of the colony (O'Donnell 1998b).

Dominance interactions in *M. consimilis* do not seem to have a direct correlation with the frequency of foraging activity as suggested by Premnath et al. (1995) and O'Donnell (1998a) or with the structuring of polyethism (O'Donnell 1998b), since the colonies have fewer workers that must accomplish different tasks from the very first days of life, such as foraging activity, which can be performed in the first week after emergence.

The colonies of *M. consimilis* do not exhibit a well—defined temporal polyethism because most of their activities are carried out throughout their entire lifespans. This pattern evidences a behavioral plasticity among the workers similar to that which occurs in colonies of *P. versicolor* (Zara and Balestieri 2000). Several studies have shown that independent—founding species have a weak or nonexistent correlation between age and tasks performed by workers (Cameron 1989; Jeanne 1991a; Giray et al. 2005). This characteristic seems to be beneficial to the survival of *Mischocyttarus* as much as *Polistes*, because both genera include species with small colonies and with independent foundation (Giannotti 1999; Giannotti and Machado 1994).

Factors such as the body size and colony composition show better correlations with the behavioral changes of individuals in the transition from intra—nest tasks to outdoor tasks such as foraging activity (Brian 1952; Free 1955; Cameron 1989; Röseler and Van Honk 1990). According to Jeanne et al. (1988), *Polybia occidentalis*, a swarm— founding wasp with large colonies, shows a clearer division of tasks according to age. Therefore, the presence of a larger number of workers in a colony allows a well—defined temporal polyethism (Jeanne et al. 1988; O'Donnell 2001). However, even in some ‘less derived’ species like a *R. marginata* (Naug and Gadagkar 1998), there may be a well—defined temporal polyethism.
